# Comparative genomics of bacteria in the genus *Providencia* isolated from wild *Drosophila melanogaster*

**DOI:** 10.1186/1471-2164-13-612

**Published:** 2012-11-13

**Authors:** Madeline R Galac, Brian P Lazzaro

**Affiliations:** 1 Field of Genetics and Development, 3125 Comstock Hall, Cornell University, Ithaca, NY, 14853, USA

**Keywords:** *Providencia*, *Drosophila melanogaster*, Whole genome comparison, Host-pathogen interactions, Genomics, *Providencia rettgeri*, *Providencia sneebia*, *Providencia alcalifaciens*, *Providencia burhodogranariea*

## Abstract

**Background:**

Comparative genomics can be an initial step in finding the genetic basis for phenotypic differences among bacterial strains and species. Bacteria belonging to the genus *Providencia* have been isolated from numerous and varied environments. We sequenced, annotated and compared draft genomes of *P*. *rettgeri*, *P. sneebia, P*. *alcalifaciens*, and *P. burhodogranariea*. These bacterial species that were all originally isolated as infections of wild *Drosophila melanogaster* and have been previously shown to vary in virulence to experimentally infected flies.

**Results:**

We found that these *Providencia* species share a large core genome, but also possess distinct sets of genes that are unique to each isolate. We compared the genomes of these isolates to draft genomes of four *Providencia* isolated from the human gut and found that the core genome size does not substantially change upon inclusion of the human isolates. We found many adhesion related genes among those genes that were unique to each genome. We also found that each isolate has at least one type 3 secretion system (T3SS), a known virulence factor, though not all identified T3SS belong to the same family nor are they in syntenic genomic locations.

**Conclusions:**

The *Providencia* species examined here are characterized by high degree of genomic similarity which will likely extend to other species and isolates within this genus. The presence of T3SS islands in all of the genomes reveal that their presence is not sufficient to indicate virulence towards *D. melanogaster*, since some of the T3SS-bearing isolates are known to cause little mortality. The variation in adhesion genes and the presence of T3SSs indicates that host cell adhesion is likely an important aspect of *Providencia* virulence.

## Background

*Providencia* are ubiquitous Gram-negative bacteria in the family *Enterobacteriaceae* that are often opportunistic pathogens. They are commonly found to cause traveler’s diarrhea and urinary tract infections in humans but have also been isolated from more severe human infections such as meningitis [1-4]. They have been identified as part of the normal human gut flora and the genomes of some strains have been sequenced as part of the Human Microbiome Project [5]. Additionally, *Providencia* have been associated with numerous animals worldwide, including isolation from penguin feces in German zoos [6], sea turtles in the Mediterranean [7], shark mouths in Brazil [8], entomopathogenic nematodes globally [9], and snakes from Vietnam [10]. *Providencia* have also been found in association with insects such as blowflies [11], stable flies [12], Mexican fruit flies [13], and house flies [14]. *Providencia* have also been found in the guts and external environment of *Drosophila melanogaster*[15,16].

*Providencia* strains have been additionally isolated as infectious agents of *D. melanogaster* and have been shown to have distinct phenotypes including varied virulence towards *D. melanogaster*[17,18]. Two species, *Providencia sneebia* and *Providencia alcalifaciens*, were found to be highly virulent, causing 90-100% mortality in infected flies. Infections with the other two, *Providencia rettgeri* and *Providencia burhodogranariea,* caused only moderate mortality, with 30-40% of infected flies succumbing to the infection [18]. The more lethal bacteria tended to proliferate to higher densities in the fly, and triggered greater expression of antibacterial immune genes, with the exception of *P. sneebia*, which did not induce substantial antimicrobial peptide gene expression despite rapid and lethal proliferation [18].

In the present work, we have sequenced, annotated, and compared the draft genomes of the four species isolated from infections in wild *D. melanogaster*: *P. sneebia, P. rettgeri, P. burhodogranariea* and *P. alcalifaciens*. We compared our sequences to draft genomes of four sequenced species of *Providencia* isolated from the human gut [5]. We sought specifically to identify the core *Providencia* genome and accessory genes, to establish which genes may be evolving under positive selection, and to annotate differences in gene content that contribute to physiological differences among the isolates.

## Methods

### Bacteria strains sequenced

The four bacteria strains that were sequenced in this study were isolated from the hemolymph of wild caught *Drosophila melanogaster*[18]. They are the *Providencia sneebia* Type strain (DSM 19967) [GenBank:AKKN00000000], *Providencia rettgeri* strain Dmel1 [GenBank:AJSB00000000], *Providencia alcalifaciens* strain Dmel2 [GenBank:AKKM00000000], and *Providencia burhodogranariea* Type strain (DSM 19968) [Genbank:AKKL00000000]. The versions described in this paper are the first versions, XXXX01000000.

### Genome sequencing and assembly of *P. rettgeri* and *P. sneebia*

Bacterial DNA was extracted using Puregene DNA Purification Kit (Gentra Systems Inc., Minneapolis, MN) according to the manufacturer’s directions for Gram-negative bacteria. The DNA was then sequenced using FLX Roche/454 Sequencing Technology at Cornell University’s Life Science Core Laboratory Center in Ithaca, NY.

*P. sneebia* and *P. rettgeri* were sequenced with approximately 500,000 reads of an average length of 250 bp, providing about 30X coverage for each genome. The sequences for each species were obtained from separate full-plate sequencing runs and independently assembled using the LaserGene SeqMan (version 8) software with the manufacturer’s recommended parameters (Additional file 1: Figure S1). An additional 180,000 reads were obtained from a 454 sequencing plate on which the DNA from *P. sneebia* and *P. rettgeri* was separated by a rubber gasket. During the sequencing process, this gasket leaked allowing a very small amount of reciprocal contamination. We did not want to discard these sequences entirely, but we also wanted to avoid any contaminating reads fouling our assemblies. Therefore our second assembly step was to assemble the reads from the half-plate to those contigs initially assembled with the uncontaminated full-plate reads using SeqMan (Additional file 1: Figure S1). From this second step of assembly, we retained: (1) contigs that contained half-plate reads assembled to full-plate contigs, increasing the depth of those contigs, (2) contigs in which half-plate reads bridged previously separate contigs from the full-plate assemblies, allowing them to be stitched together, and (3) novel contigs containing only half-plate reads but with a coverage depth of 30X or greater. Contaminating sequences in the half-plate reads would presumably fail to map to full-plate assemblies or would result in low-coverage contigs, so we infer that the small number of molecules that leaked through the gasket have been effectively discarded. After the second round of assembly, the *P. sneebia* genome was mapped into 72 contigs and the *P*. *rettgeri* genome was mapped into 71 contigs.

As we were annotating the *P*. *sneebia* and *P*. *rettgeri* genome sequences (see “Annotation methods” section below), we noticed several instances of sequential open reading frames (ORFs) that were annotated with the same predicted function and whose combined length equaled the size of genes with the same functional annotation in other bacteria. Closer inspection revealed that these instances were generally due to a stop codon or frameshift mutation that interrupted the ORF, causing it to be annotated as two genes with identical function. These inferred mutations tended to happen after short homopolymer runs. Individual reads varied in the lengths of these homopolymer sequences, and the contig assembly often did not reflect the most common sequence length among the reads. It is a known problem that Roche/454 Sequencing often results in errors in homopolymer run lengths [19]. To improve the accuracy of inferred homopolymer lengths, we re-aligned all of the Roche/454 sequencing reads to our assembled reference sequences using the program BWA [20] (Additional file 1: Figure S1). The consensus homopolymer length from the BWA alignment was used to fix the assembled contigs before any further analysis was performed. This correction improved our gene annotations by eliminating sequencing errors that interrupted ORFs.

We aimed to improve the assemblies of our *P*. *sneebia* and *P*. *rettgeri* genomes by joining contigs through PCR followed by direct Sanger sequencing. However, the order and orientation of the contigs was unknown. We hypothesized that there would be synteny among the genomes of *Providencia* species and isolates as well as species in the closely related genus *Proteus*, which we could use to predict the order and orientations of the contigs in our assemblies (Additional file 1: Figure S1). We used MUMmer (version 3.22) [21] to compare *P. sneebia* and *P. rettgeri* to the draft genomes of *Providencia rettgeri* DSM 1131 (283 contigs) [GenBank:ACCI00000000], *Providencia alcalifaciens* DSM 30120 (79 contigs) [GenBank:ABXW00000000], *Providencia stuartii* ATCC 25827 (120 contigs) [GenBank:ABJD00000000], and *Providencia rustigianii* DSM 4541 (127 contigs) [GenBank:ABXV00000000] as well as the completed genome of *Proteus mirabilis* strain HI4320 [GenBank:NC_010554.1]*.* Where two of our *P. rettgeri* or *P. sneebia* contigs had similarity to a single contig of one of the other genome sequences, we designed PCR primers to amplify across the inferred gap. Successful amplifications were sequenced by primer walking and the resultant sequences were used to bridge contigs in the assemblies. PCR and sequencing methods are described below in the “PCR and Sanger sequencing methods” section. We found that designing the primers inset about 900 bp from the contig breakpoints helped to ensure specificity in amplification, especially because repetitive sequences in the genome can be the cause of contig breaks in genome assemblies. Using this method, we reduced the number of contigs in the *P. sneebia* assembly from 72 to 67 and in the *P. rettgeri* assembly from 71 to 64.

To further connect the *P. sneebia* and *P. rettgeri* assemblies, we contracted the MapIt optical mapping service from OpGen, Inc. (Gaithersburg, MD) and analyzed the resulting data using their program MapSolver (version 2.1.1) (Additional file 1: Figure S1). An *in silico* digestion of our contigs allowed them to be oriented onto an *in vitro* restriction digestion map of each bacterium’s physical genome. This ordered and oriented the contigs and allowed us to identify those contigs that comprised the majority of each genome. We used the optical map to identify physically consecutive contigs, then designed primers for PCR and Sanger sequencing to close most of the remaining gaps. The optical map also indicated a small number of computational misassemblies and allowed them to be fixed. After optical mapping and final gap closing, our draft genome sequences were assembled into 14 contigs for *P. sneebia* and 9 contigs for *P. rettgeri.*

### Genome sequencing and assembly of *P. alcalifaciens* and *P. burhodogranariea*

*P. alcalifaciens* and *P. burhodogranariea* were sequenced by paired-end 454 sequencing. Libraries were constructed for each bacterium with an approximately 3 kb insert size, and roughly 1 million paired-end sequence reads of an average length of 250 bases were collected. The paired-end reads were assembled using Roche/454’s Newbler Assembler (version 2.5.3), which resulted in 15 scaffolds for *P. alcalifaciens* and 8 scaffolds for *P. burhodogranariea*, sequenced to roughly 35X coverage.

### PCR and Sanger sequencing methods

PCR primers for gap closing were designed either using Primer3 [22] or with a primer design function within SeqMan. PCRs were performed using a genomic DNA template with a final concentration of 1.2 ng/μl in each PCR reaction volume.

When the size of the expected product was unknown or was expected to be less than 5 kb, the PCR was done with Taq polymerase (New England Biolabs, Beverly, MA) with an annealing temperature gradient ranging from 2°C higher to 2°C lower than the melting temperature of the primers. PCR cycling parameters were as follows: (1) 2 minutes at 95°C, (2) 30 seconds at 95°C, (3) 30 seconds at annealing temperature gradient, (4) 1 minute at 72°C, (5) repeat steps 2–4 for 34 more cycles, (6) 5 minutes at 72°C. 3.5 μl of each PCR product was prepared for sequencing by treatment with 5 units of Exonuclease I (USB Corp., Cleveland, OH) and 0.5 units of shrimp alkaline phosphatase (USB Corp., Cleveland, OH) at 37°C for one hour before heat-killing the enzymes at 65°C for 15 minutes. PCR products were then directly sequenced using ABI BigDye Terminator (Applied Biosystems, Foster City, CA) according to the manufacturer’s directions.

PCR for products with an expected size greater than 5 kb was done using high fidelity iProof polymerase (Bio-Rad, Hercules, CA). Annealing temperatures and extension times were determined using manufacturer’s suggested methods. The PCR cycling parameters were: (1) 30 seconds at 98°C, (2) 7 seconds at 98°C, (3) 20 seconds at appropriate annealing temperature, (4) appropriate extension time at 72°C, (5) repeat steps 2–4 for 29 more cycles, (6) 7 minutes at 72°C. Products were prepared for sequencing with PCR purification clean up columns (Invitrogen, Calsbad, CA) before being sequenced directly.

### Annotation methods

Genomic open reading frames were determined and annotated using the RAST Server (version 4) [23]. Gene ontology terms (GO terms) were assigned to the ORFs identified by RAST using Blast2GO (version 2.5) [24]. Fisher’s Exact Test for enriched GO categories was done within Blast2GO using a *p*-value cut off of 0.05 after adjusting for a standard false discovery rate (FDR) of 0.05 for multiple testing.

### Plasmid identifications and analysis

The circular DNA structure of plasmids means that they will appear to be arbitrary broken when forming linear contigs during assembly of sequencing reads. We tested all potential plasmid contigs for a circular physical structure by designing PCR primers approximately 500 bases from the ends of the contig facing outward off each end of the contig. This primer design means that a product would be formed only if the ends of the contig were connected in the physical DNA. Any PCR product amplified from such primers was then sequenced with Sanger sequencing to confirm that the sequence supported a circular physical arrangement of the sequence. PCR reactions and Sanger sequencing was done as described above in “PCR and Sanger sequencing methods”.

Putative plasmids were identified in multiple ways. We speculated that one *P. rettgeri* contig might be a plasmid because it had substantially higher depth of coverage than other contigs, and when we compared the contig to itself using MUMmer, we found that it was composed of the same sequence repeated multiple times. We hypothesized that the contig might actually represent a completely sequenced, high copy number plasmid, and that the circular shape of the physical DNA sequence was resulting in a tandem repeat of the sequence in the *in silico* assembly. PCR and Sanger sequencing confirmed that this contig is a plasmid.

To more systematically assess whether contigs from the assemblies were plasmids, we looked for contigs with identical sequence present at both ends. We hypothesized that the arbitrary break point of the physical circular structure to form a linear contig could result in identical sequence at each end of the contig. We used MUMmer to compare contigs to themselves. For *P. sneebia* and *P. rettgeri,* we examined all contigs which did not align to the optical map since we do not expect plasmids to map to the chromosomal genome. We identified three *P. sneebia* plasmids using this method, all of which were confirmed by PCR and Sanger sequencing. We identified no additional *P. rettgeri* contigs as putative plasmids using this approach. For *P. alcalifaciens* and *P. burhodogranariea*, we examined every scaffold smaller than 6 kb in length, but none contained the same sequence at both ends of the contig. It should be noted that this method may result in an underestimation of plasmids since it will not detect plasmids that assemble into multiple contigs.

When examining the synteny of the genomes (see “Synteny and regional comparisons” section below), we noticed that some of the contigs of *P. alcalifaciens* had no similarity to sequences in any of the other genomes. We hypothesized that these contigs could be plasmids that are unique to *P. alcalifaciens.* We tested four contigs by PCR and Sanger sequencing. One contig was confirmed to be a plasmid while the other three did not produce PCR products and therefore showed no evidence of being plasmids.

All putative plasmids were compared to each other using MUMmer. In order to determine whether our confirmed plasmids or previously sequenced *Providencia* plasmids [25-27] were present but undetected in the remaining sequences from this study, we constructed a BLAST database of all of the reads from the Roche/454 sequencing of each individual species and searched for reads matching each *Providencia* plasmid using BLAST+ (version 2.2.25). Four previously identified *Providencia* plasmids were used as query sequences: pDIJ09-518a [GenBank:HQ834472.1], pGHS09-09a [GenBank:HQ834473.1], pMR0211 [GenBank:JN687470.1], and R7K [Genbank:NC_010643.1].

### Identification of orthologs

Orthologous genes were identified as shared among three different sets of bacteria: (1) the strains of *P. sneebia, P. rettgeri, P. alcalifaciens,* and *P. burhodogranariea* sequenced in this study; (2) the strains of *P. sneebia, P. rettgeri, P. alcalifaciens,* and *P. burhodogranariea* sequenced in this study plus the strains of *P. rettgeri, P. alcalifaciens, P. stuartii,* and *P. rustigianii* sequenced as part of the Human Microbiome Project [5]; (3) the strains of *P. sneebia, P. rettgeri, P. alcalifaciens,* and *P. burhodogranariea* sequenced in this study plus the strains of *P. rettgeri, P. alcalifaciens, P. stuartii,* and *P. rustigianii* sequenced as part of the Human Microbiome Project plus the outgroup *Proteus mirabilis* strain HI4320. Orthologous gene clusters were identified using OrthoMCL (version 2.0.2) [28]. BLAST results used within OrthoMCL were performed with an e-value cut off of 10^-10^. The output from OrthoMCL was parsed using custom Python scripts.

### Alignments of orthologs

Orthologous gene clusters found among the strains sequenced in this study, those *Providencia* sequenced as part of the Human Microbiome Project, and *Proteus mirabilis* were aligned for phylogenetic analysis (see “Phylogenetic analysis” section below). Those orthologs of the strains sequenced in this study only were aligned for use in the recombination and positive selection analyses (see “Recombination analysis” and “Positive selection analysis” sections below). Only clusters with clear single gene orthology across each genome were retained. Alignments of the protein translation of the genes were done using ClustalW (version 2.1) [29] followed by back-translation to the nucleotide alignment using PAL2NAL (version 13) [30]. Alignments were visually inspected and poor alignments were removed as follows. We eliminated alignments where the difference in amino acid identity between the most-similar and least-similar pairs of species were greater than 60% out of concern that these might not be true orthologs. We also excluded alignments that had both an average protein identity that was less than 65% and a difference between the highest and lowest pair-wise protein identities greater than 35%. Those alignments that had an average protein identity of less than 75% were examined by hand to ensure proper alignment.

### Phylogenetic analysis

The alignments of all 1651 ortholog clusters that included all eight sequenced *Providencia* genomes and *Proteus mirabilis* were concatenated using FASconCAT (version 1) [31]. RAxML (version 7.2.8) [32] was used to construct the phylogenetic trees for the concatenation of all orthologous genes. *Proteus mirabilis* was set as an outgroup.

### Synteny and regional comparisons

Synteny among genomes was examined using Mauve (version 2.3.1) [33], Artemis Comparison Tool (version 1) [34], and MUMmer. Comparisons of particular regions of the genomes were done using EasyFig (version 1.2) [35].

### Recombination analysis

Evidence for recombination was examined by executing the programs GENECONV which implements the Sawyer method [36] and PhiPack [37]. GENECONV was run using the default settings, which estimates *p-*values on 10,000 permutations of each alignment. PhiPack runs 3 separate tests: Pairwise Homoplasy Index, Maximum χ^2^, and Neighbor Similarity Score. The Pairwise Homoplasy Index was calculated on a window size of 50 while Maximum χ^2^ was calculated on a window that is 2/3 the size of the polymorphic sites. We did 1000 permutations in PhiPack to calculate each *p*-value. The *p*-values of all tests were corrected for multiple testing using the program Q-value [38] with a FDR of 10%, acknowledging that the multiple tests conducted are highly intercorrelated and a stricter correction would therefore be overly conservative [39,40].

### Positive selection analysis

Positive selection analysis was done using PAML (version 4.4) [41] on the 1937 orthologous clusters found in the *Providencia* sequenced in this study. Site-model tests were implemented using codeml to compare model M8a (beta+ω=1) to M8 (beta+ω) [41,42]. The log-likelihoods from each test were compared in a likelihood ratio test assuming a χ^2^ distribution of the test statistic. RAxML [32] was used to construct the phylogenetic trees for each ortholog cluster examined in this analysis. We corrected for multiple testing using a q-value cut off which was calculated with the program Q-value [38] using a FDR of 20%, reflecting the conservative nature of the positive selection test [39,40].

### Phage identification

Phage genes were identified and classified using PHAST [43].

### Predicting the core genome size

Custom scripts were written in Python to determine the number of orthologs for all combinations of 2, 3, 4, 5, 6, or 7 genomes of the eight sequenced *Providencia* genomes by parsing the OrthoMCL output described in the “Identification of orthologs” section.

## Results and discussion

### Basic genome information

**Figure 1:**
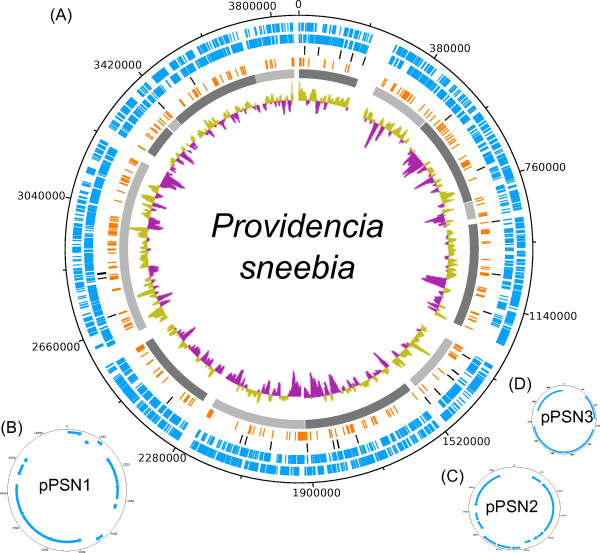
**Circular maps of the *Providencia sneebia *genome and plasmids.** (**A**) *P. sneebia* genome. The contigs are ordered and oriented as they are in the physical genome based on an optical map, including the sizes of the gaps between contigs. Rings from the outermost to the center: 1) genes on the forward strand (blue), 2) genes on the reverse strand (blue), 3) tRNA and rRNA genes (black), 4) genes unique to *P. sneebia* when comparing all 8 Dmel and HMP *Providencia* genomes (orange), 5) individual assembly contigs (alternating shades of grey), 6) GC skew. (**B**) pPSN1. (**C**) pPSN2. (**D**) pPSN3. All plasmids have the genes on the forward strand on the outermost ring and genes on the reverse strand on the inner ring.

**Figure 2:**
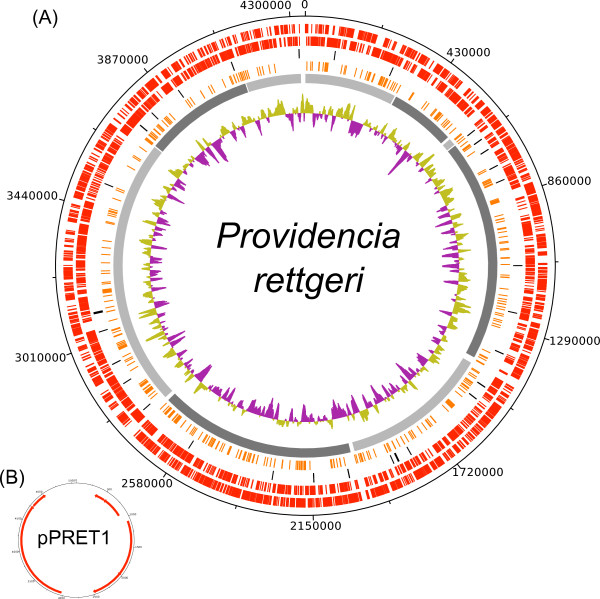
**Circular maps of the *Providencia rettgeri *genome and plasmid.** (**A**) *P. rettgeri* genome. The contigs are ordered and oriented as they are in the physical genome based on an optical map, including the sizes of the gaps between contigs. Rings from the outermost to the center: 1) genes on the forward strand (red), 2) genes on the reverse strand (red), 3) tRNA and rRNA genes (black), 4) genes unique to *P. rettgeri* when comparing all 8 Dmel and HMP *Providencia* genomes (orange), 5) individual assembly contigs (alternating shades of grey), 6) GC skew. (**B**) pPRET1. The plasmid has the genes on the forward strand on the outermost ring and genes on the reverse strand on the inner ring.

**Figure 3:**
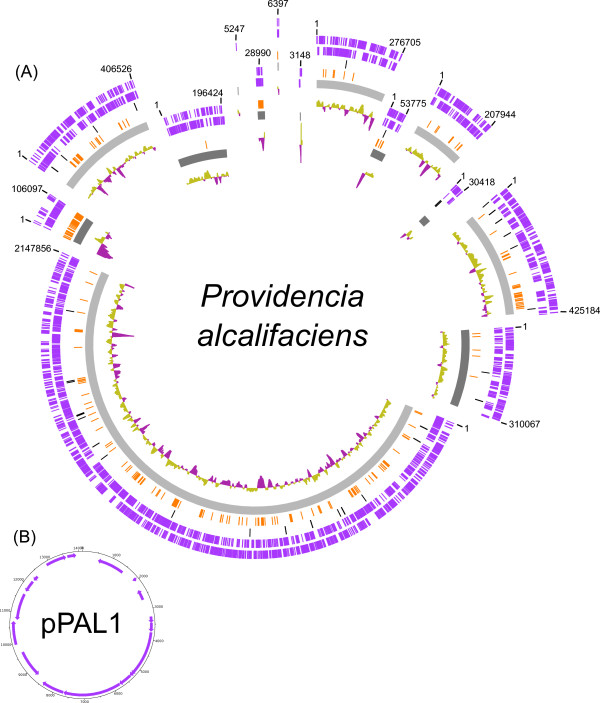
**Maps of the *Providencia alcalifaciens *genome and plasmid.** (**A**) *P. alcalifaciens* genome. The scaffolds are ordered and oriented for maximum synteny with *P. rettgeri*. The scaffolds are not positioned in a complete circle because the order and orientation of the scaffolds are not empirically known. The size of the gaps between scaffolds is unknown. Rings from the outermost to the center: 1) genes on the forward strand (purple), 2) genes on the reverse strand (purple), 3) tRNA and rRNA genes (black), 4) genes unique to *P. alcalifaciens* when comparing all 8 Dmel and HMP *Providencia* genomes (orange), 5) individual scaffolds (alternating shades of grey), 6) GC skew. (**B**) pPALC1. The plasmid has the genes on the forward strand on the outermost ring and genes on the reverse strand on the inner ring.

**Figure 4:**
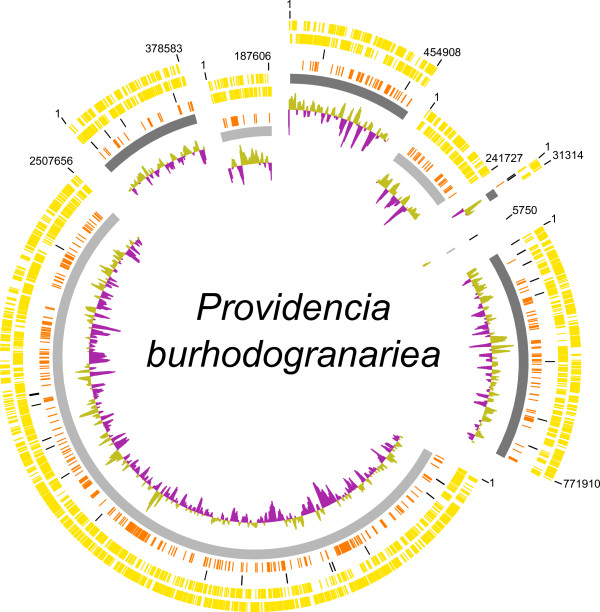
**Map of the *Providencia burhodogranariea *genome.** The scaffolds are ordered and oriented for maximum synteny with *P. rettgeri*. The scaffolds are not positioned in a complete circle because the order and orientation of the scaffolds are not empirically known. The size of the gaps between scaffolds is unknown. Rings from the outermost to the center: 1) genes on the forward strand (yellow), 2) genes on the reverse strand (yellow), 3) tRNA and rRNA genes (black), 4) genes unique to *P. burhodogranariea* when comparing all 8 Dmel and HMP *Providencia* genomes (orange), 5) individual scaffolds (alternating shades of grey), 6) GC skew.

The genomes of *Providencia sneebia, Providencia rettgeri, Providencia alcalifaciens* and *Providencia burhodogranariea* were sequenced in this study and assembled into 14, 9, 15 and 8 contigs or scaffolds*,* respectively (Table 1, Figures 1, 2, 3, and 4)*.* The sequenced isolates were obtained from the hemolymph of wild *Drosophila melanogaster* and therefore will be referred to collectively as Dmel isolates in this paper. The summed contig or scaffold lengths of these assemblies vary from 4.5 Mb to 3.5 Mb, with *P. burhodogranariea* having the largest genome and *P. sneebia* having the smallest (Table 1). Because we know the sizes of the *P. sneebia* and *P. rettgeri* physical genomes from optical maps, we can discern that our *P. sneebia* assembly is missing approximately 300 kb of sequence and our *P. rettgeri* assembly is missing approximately 100 kb of sequences (Table 1). Based on the average gene size for each genome, we estimate that our annotated gene sets are missing roughly 275 out of 3750 for *P. sneebia* and 107 genes out of 4650 total for *P. rettgeri* (Table 1). Thus, we estimate that we have assembled approximately 93% of the *P*. *sneebia* coding genome and 98% of the *P. rettgeri* coding genome. Both *P. alcalifaciens* and *P. burhodogranariea* were sequenced with paired-end reads, resulting in scaffolds with sequence gaps of known sizes. These gaps can break up open reading frames, resulting in the absence of some genes from our inferred annotations. Since we did not optically map *P. alcalifaciens* or *P. burhodogranariea*, we do not know the precise sizes of their physical genomes. Because of the small assembly gaps, our annotations bear the caveat that a small number of genes may be inferred as absent when they are actually present in the physical genome.

**Table 1:** **Basic genomic information of all four *D****.****melanogaster *isolated *Providencia***

**Species**	**Strain**	**DSM**^**1**^		**Sequenced size**^**2**^	**Physical size**^**3**^	**# of contigs or scaffolds**	**Average GC %**	**# of genes**	**Est. # of missing genes**^**4**^
*Providencia sneebia*	Type	19967	chromosome	3.5 Mb	3.8 Mb	14	38.08	3482	275
			pPSN1	10787 bp	10787 bp	1	33.50	12	0
			pPSN2	7592 bp	7592 bp	1	35.05	12	0
			pPSN3	4321 bp	4321 bp	1	31.50	4	0
*Providencia rettgeri*	Dmel1	-	chromosome	4.2 Mb	4.3 Mb	9	40.20	4532	107
			pPRET1	5567 bp	5567 bp	1	40.81	6	0
*Providencia alcalifaciens*	Dmel2	-	chromosome	4.2 Mb	unk	15	41.17	3900	unk
			pPALC1	14114 bp	14114 bp	1	37.57	17	0
*Providencia burhodogranariea*	Type	19968	chromosome	4.5 Mb	unk	8	38.23	3985	unk

^1^Strain number in DSM Culture Collection.

^2^Sum of the contigs or scaffolds lengths.

^3^Size of the physical chromosome as determined by optical maps. “unk” indicates unknown physical size.

^4^Estimated from the known length of sequence missing from these assemblies divided by the average gene length. “unk” indicates that the number of missing genes cannot be calculated due to an unknown physical size.

### Plasmids

We found 3 plasmids in *P. sneebia,* 1 in *P. rettgeri*, 1 in *P. alcalifaciens* and none in *P. burhodogranariea* (Table 1; Figures 1, 2, 3, and 4)*.* We compared these plasmids to each other and found them to have no similarity in gene content. We also checked whether any of these plasmids were undetected in the sequencing reads of the Dmel isolates other than the one that each plasmid was assembled from. We found that while some genes present on these plasmids were similar to paralogs on the chromosomes of other species, the complete plasmids were found only in the species from which they were initially recovered. Four plasmids have been isolated and sequenced by other groups studying other *Providencia* isolates [25-27], but we did not find any of these plasmids in the sequencing reads of our isolates.

We examined the plasmids for the presence of replication genes in order to determine their incompatibility group. The *repA* gene of pPALC1 has an IncFII domain and is most similar to one found in *Yersinia pseudotuberculosis.* This incompatibility group indicates that the plasmid has a host range limited to *Enterobacteriaceae*[44]. We found pPRET1 to have a Rop protein, which is similar to one found in *Proteus vulgaris,* suggesting that it uses an RNA based method of replication [45]. The replicase present on pPSN3 was found to contain a PriCT-1 domain, which has been found in a wide variety of bacteria, bacteriaphages and plasmids [46]. This replicase was similar to those found in numerous *E. coli* strains. We were unable to identify the replicase proteins for pPSN1 and pPSN2. This is most likely due to them using a replication method which is not well characterized. Plasmids previously isolated from different *P. rettgeri* isolates also do not carry obvious replicase genes [25], and pPSN1 and pPSN2 do not show similarity to these plasmids so it is likely that at least three different mechanisms are being used.

None of our novel *Providencia* plasmids contain genes with functional annotations that lead to a clear functional designation for the overall plasmid, such as “virulence” or “antibiotic resistance.” Our findings indicate that plasmids found in *Providencia* vary considerably in their identity, conservation, replication methods and probable functions.

### Genomic synteny

**Figure 5:**
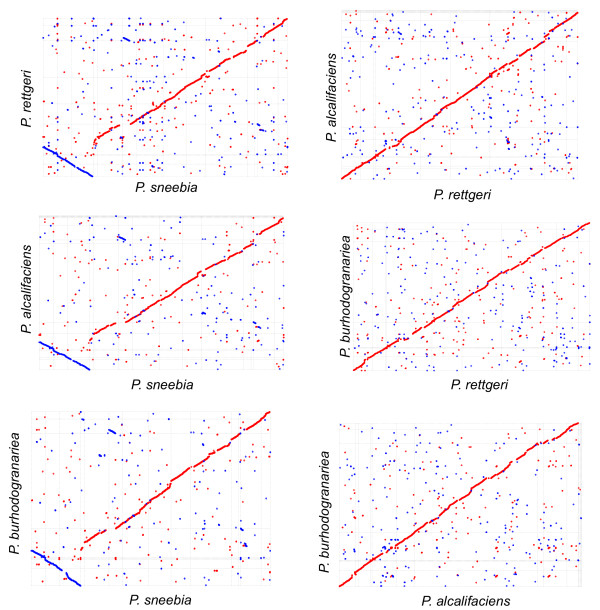
**Alignments of the protein translations of the whole genomes of all four *Providencia *species isolated from *D. melanogaster.*** The contigs of *P. sneebia* and *P. rettgeri* were ordered and oriented as they are in the physical genome based on optical maps made of each genome. *P. alcalifaciens* and *P. burhodogranariea* contigs were ordered and oriented for maximum synteny with *P. rettgeri*, therefore assuming parsimony in the number of genomic arrangements*.* Similarity was calculated using promer function in MUMmer [21]. Red dots represent similar sequence in the same orientation in each genome pair while blue indicates that the similarity is in the opposite orientations in the genome pairs.

The optical maps of *P. sneebia* and *P. rettgeri* allowed us to order and orient the contigs as they are found in the physical chromosome for those two assemblies*.* The contigs of *P. burhodogranariea* and *P. alcalifaciens* were ordered and oriented so that they were as similar to the *P. rettgeri* and *P. sneebia* genomic orientations as possible, assuming the most parsimonious evolution of genome arrangements (Figure 5). It is in principle possible that any of the *P. burhodogranariea* and *P. alcalifaciens* contigs could be inverted or rearranged relative to their positions on our comparative syntenic plot, but only if the rearrangement breakpoints lie at contig breakpoints. While there are many small rearrangements found among the genomes, there are only two large inversions apparent across the four species. Both inversions are in *P. sneebia* relative to the other genomes (Figure 5). The ends of the largest *P. sneebia* inversion, which is about 800 kb in length, fall within single contigs of both *P. burhodogranariea* and *P. alcalifaciens*, supporting the hypothesis that the inversion is derived in and unique to *P. sneebia*.

### Phylogeny

**Figure 6:**
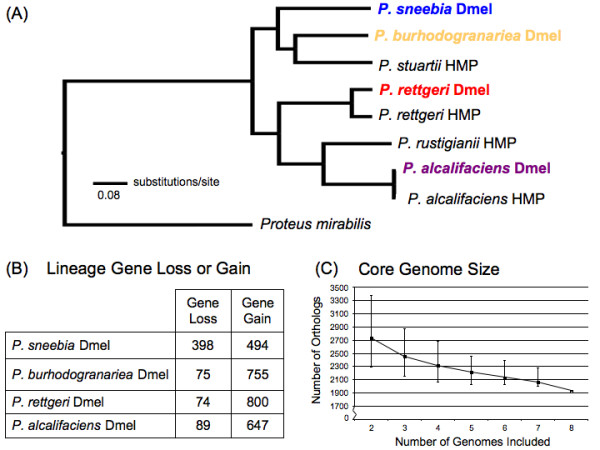
**(A) Phylogenetic relationships of eight sequenced *Providencia *strains and the outgroup *Proteus mirabilis.*** This phylogeny is constructed from a concatenation of 1651 orthologous genes shared by all nine genomes and was inferred using maximum likelihood methods within the program RAxML [32]. The *Providencia* isolated from *D. melanogaster* are shown in bold and color. The scale bar indicates number of substitutions per site. Each node of the tree is supported by a bootstrap value of 100. (**B**) Gene losses and gains along lineages leading to *Providencia* isolated from *D. melanogaster*. Numbers indicate the number of inferred gene gains and losses on each lineage for the four strains isolated from *D. melanogaster*. These numbers highlight that *P. sneebia* has experienced a much greater gene loss than the other species. (**C**) Core genome size when different numbers of genomes are included. The point indicates the average number of orthologs in each comparison while the error bars indicate the highest and lowest number of orthologs for all possible combinations of each number of genomes.

We determined an overall phylogenetic relationship from a concatenated alignment of 1651 single-copy orthologs shared by all four Dmel *Providencia* sequenced here, rooted with *Proteus mirabilis* (Figure 6A). The phylogenetic tree indicates that *P. sneebia* and *P. burhodogranariea* share a common ancestor before either of them share a common ancestor with the *P. rettgeri* and *P. alcalifaciens* species pair (Figure 6A). This is consistent with the phylogenetic relationships proposed previously based on the 16S rRNA gene and five housekeeping genes [17].

### Orthologs and unique genes

**Figure 7:**
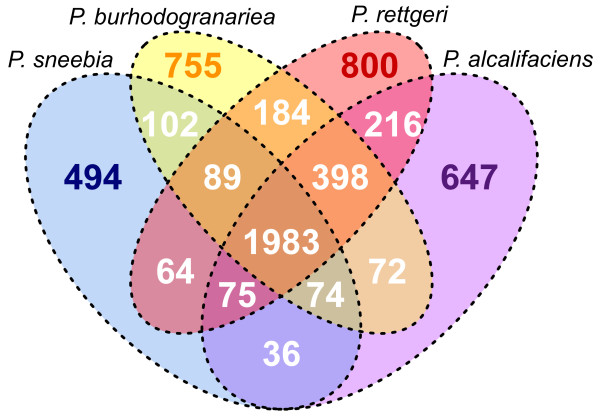
**Shared and unique gene totals among all four *Providencia *species isolated from *D. melanogaster.*** Numbers are the gene counts within each sector of the Venn diagram. Orthologous genes were determined with OrthoMCL [28].

We wanted to know how much gene content is shared among the Dmel *Providencia* isolates so we used a BLAST-based method to find all orthologous gene clusters. There are a total of 3644 orthologous clusters containing between 10 and 2 genes, as well as one exceptional cluster containing 20 fimbrial-related usher genes with at least 3 genes originating from each genome. Fimbrial-related usher proteins chaperone other proteins to the bacterial cell surface to form a proteinaceous extension involved in surface adhesion [47]. We found that 3293 genes are present as single copies in each genome, or 90% of the total ortholog clusters. The majority of these clusters, 1983, comprise the core genome of these *Providencia* isolates (Figure 7), meaning that the genes are shared as single-copy orthologs across all four sequenced species. The core genome is 49-62% of the total genes in each genome, revealing that the species have a substantially homogeneous gene content. The next largest group of orthologous clusters is that which contains genes present in *P. rettgeri, P. alcalifaciens,* and *P. burhodogranariea* but absent from *P*. *sneebia* (Figure 7)*.* This is consistent with these three genomes each being almost one megabase bigger than that of *P. sneebia.* Given that *P*. *sneebia* shares a common ancestor with *P. burhodogranariea* before either shares a common ancestor with the more distantly related *P. rettgeri* and *P*. *alcalifaciens* (Figure 6A), the absence of these genes in *P*. *sneebia* appears to reflect genome reduction in *P. sneebia*. To specifically examine putative gene loss, we determined the number of orthologous clusters that were missing a gene from only 1 of the 4 genomes (Figures 6B and 7). While *P. rettgeri, P. alcalifaciens,* and *P. burhodogranariea* have between 74 and 89 genes that are apparently lost in their lineages, *P. sneebia* appears to have specifically lost 398 genes. As revealed in the analysis of genomic synteny above, differences in gene number and genome sizes generally are the result of small duplications, deletions, or insertions. In particular, the missing genes in *P*. *sneebia* do not result from large block deletions but instead arise from many small deletions eliminating individual genes distributed around the genome (Figure 5).

We found 137 orthologous clusters which contained paralogous duplications unique to a single genome. The majority of these clusters contain genes that are related to mobile elements or phages suggesting that there are families of transposons or phages that are specific to individual species. Most other clusters of species-specific paralog groups were annotated as hypothetical proteins.

We were able to determine genes unique to each genome by identifying those genes that were not assigned to any orthologous cluster (Figures 6B and 7). The genomes varied in the absolute number of unique genes, with the smallest genome, that of *P. sneebia,* having the fewest. Despite their variation in number among the genomes, unique single-copy genes represent 15-19% of their total genome content for each species.

**Figure 8:**
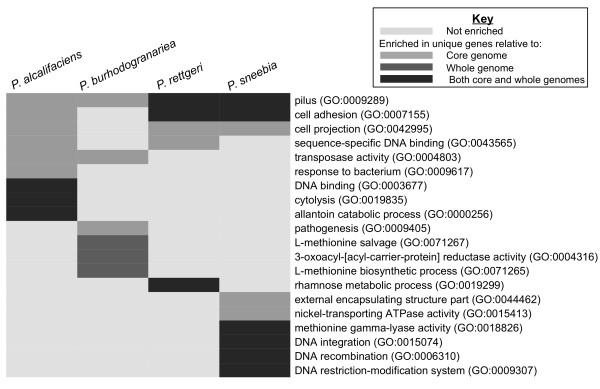
**GO terms enriched in the unique genes of each species compared to the full set of *Providencia *isolated from *D. melanogaster.*** GO terms were assigned and calculation of enrichment was done using a Fisher’s Exact Test using Blast2GO [24]. GO terms have been collapsed to only the most specific child term when multiple terms described the same group of genes.

The genes unique to each bacterium were tested for enrichment of gene ontology (GO) terms compared to those genes found in either the Dmel *Providencia* core genome or the individual whole genome from which the unique genes were drawn (Figure 8). GO terms enriched among the genes unique to each species were often related to interactions with phage or bacteria, including genes encoding phage lysozymes, bacteriocins, and restriction enzymes. This strongly suggests that these *Providencia* have acquired or developed different ways to deal with varied genome parasites and competitor organisms. This may also imply variation in the phage or bacteria to which these *Providencia* are most often exposed.

Based on GO annotation, the genes unique to *P. rettgeri* are enriched for those involved in rhamnose metabolic process compared to both the core and whole genome. While the particular strain of *P. rettgeri* we sequenced has not been tested for its ability to metabolize rhamnose, the type strain of *P. rettgeri* has been shown to metabolize this sugar while the same strains of *P. sneebia* and *P. burhodogranariea* sequenced in this study were unable to [17]. The type strain of *P. alcalifaciens* has also been tested for its ability to metabolize rhamnose, but the data were ambiguous [17]. The collective data are suggestive that rhamnose metabolism may be unique to *P. rettgeri* among these strains of *Providencia,* although previous work has shown that not all strains of *P. rettgeri* are able to metabolize rhamnose [48].

All four species have genes with the GO term “pilus” significantly overrepresented in their unique genes compared to the core genome, although the absolute number of these genes varies for each genome (Figure 8). Pili are protein structures that extend from the surface of bacterial cells to allow the bacteria to adhere to substrates [47]. The majority of the unique genes given the pilus GO term are annotated as fimbrial proteins, which are the proteins that constitute the pilus structure [47]. The genic diversity indicates a high amount of variation in these fimbrial proteins. Pilus related proteins are often antigenic, so this variation in pilus protein could be a result of pressure to avoid host immune responses [49]. Alternatively, the distinction in fimbrial proteins could be due to variation among the species in adherence to specific surfaces [49]. As mentioned above, the largest orthologous gene cluster shared among the sequenced *Providencia* consisted of fimbrial-related usher proteins. These observations in combination show conservation of the genes which function to form the pilus but diversity of the genes encoding the proteins of the physical pilus structure itself.

### Recombination and positive selection

Positive selection and recombination are two primary forces in bacterial evolution. Recombination rates have been found to vary widely in bacteria and it has been hypothesized that generalist bacteria or those in the process of adapting to new environments have higher rates of recombination [50]. These *Providencia* are closely related and were isolated from similar environments, so it is possible that there would have been opportunity for recombination among them. We examined 1937 orthologs in the core genome of the Dmel *Providencia* species for recombination, which is slightly reduced from the total 1983 core genome orthologs shown in Figure 7 due to the removal of comparatively poor sequence alignments (see Methods section “Alignments of orthologs”). Among the 1937 orthologs examined, 781 orthologous clusters that showed evidence for recombination. The genes belonging to clusters exhibiting recombination are evenly distributed around the physical genomes of each bacteria, and we do not find evidence for hotspots of recombination.

We used the program PAML [41] to test for evidence of positive selection in the *Providencia* core genome, excluding genes that showed evidence of recombination since these violate the assumptions of the tests in PAML [51]. This left 1156 orthologous clusters in the core genome shared by the four Dmel *Providencia* species isolates. We used PAML to compare the likelihood of a model which does not allow for positive selection, termed model M8a, to a model that does allow for selection at various sites along the gene, model M8 [41,42]. We found 21 genes that yielded nominal *p*-values of less than 0.05, indicating that the model allowing for selection fit the data significantly better that the neutral model (Table 2). However, none of these 21 genes remained significant after application of a FDR of 20% [38]. Our selection test is extremely underpowered within *Providencia* given the small number of species examined and their close phylogenetic relationship to one another. We considered running the same site model tests on the four Dmel *Providencia* species plus *Proteus mirabilis*, but we there are too many synonymous changes on the lineage leading to *Proteus mirabilis* for the tests to be conducted appropriately. We present some genes that show nominal evidence for positive selection below, but stress that the selection results are provisional and further investigation into the biological function and adaptive significance of these genes is warranted.

**Table 2:** Orthologous gene clusters with evidence for positive selection

**Annotation**	**SEED Subsystem**^**1**^	**EC**^**2**^	***p***-**value**
homocysteine methyltransferase	methionine biosynthesis	2.1.1.14	0.000496
xanthine/uracil/thiamine/ascorbate permease family protein	purine utilization	-	0.002595
TolC precursor	multidrug efflux pumps	-	0.003340
acetate permease ActP	acetogenesis from pyruvate	-	0.003703
hypothetical protein with DUF177	-	-	0.007188
2-octaprenyl-6-methoxyphenol hydroxylase	ubiquinone biosynthesis	1.14.13.-	0.009478
UDP-N-acetylglucosamine	-	2.4.2.227	0.010464
D-tyrosyl-tRNA(Tyr) deacylase	stringent response	-	0.014392
sulfate transport system permease protein CysT	cysteine biosynthesis	-	0.014526
yihD	-	-	0.015702
antibiotic biosynthesis monooxygenonase	-	-	0.015830
4-diphosphocytidyl-2-C-methyl-derythritol kinase	isoprenoid biosynthesis	2.7.1.148	0.018713
Acyl-phosphate:glycerol-3-phosphate O-acyltransferase PlsY	glycerolipid and glycerophospholipid metabolism	-	0.021835
dethiobiotin synthetase	biotin biosynthesis	6.3.3.3	0.029052
LSU ribosomal protein L9p	ribosomal LSU	-	0.029278
Ribonuclease E	ribosomal biogenesis, RNA processing & degradation protein glycerolipid and glycerophospholipid metabolism	3.1.26.12	0.030756
acyl carrier		-	0.031606
predicted Fe-S oxidoreductase	-	-	0.041952
flagellar transcription activator FlhC	flagellum	-	0.044270
magnesium transporter	-	-	0.048181

*P*-values were calculated using codeml within PAML [41] by comparing models M8a and M8 and are uncorrected for multiple testing.

^1^The subsystem and protein function designation were determined through annotation with RAST [23] with the exception of the proteins annotated as antibiotic biosynthesis monooxygenonase which was designated by Blast2GO [24].

^2^Enzyme Commission number.

One gene exhibiting evidence for positive selection is the *TolC* precursor (*p* = 0.003). The *TolC* gene has also been found to be under positive selection in *E. coli*[52]. This gene encodes an outer membrane protein that is part of a transporter system which transports toxins or antibiotics out of the cell [53,54]. It is possible that different proteins or varying amounts of proteins are transported by TolC in the various *Providencia* species, in which case selection may act on *TolC* to optimize interaction with different partners or vary secretion amounts.

Two different orthologous gene clusters showing weaker signal for positive selection were annotated as being involved in glycerolipid and glycerophospholipid metabolism (*p* = 0.022 and *p* = 0.032). This subsystem is involved in making lipids that are transported to the bacterial cell surface. The signatures of positive selection suggest possible adaptation in lipid and protein structures on the surface of these *Providencia* species.

We found the protein FlhC to also show weak evidence for positive selection (*p* = 0.044). This protein forms a heterodimer with FlhD to become the master regulatory complex of flagellar protein production, and these proteins have also been shown to regulate the expression of many other genes, including virulence genes [55-57].

### Similarity of Dmel isolates to *Providencia* isolates from the human gut

Four *Providencia* isolated from human feces have been sequenced as part of the Human Microbiome Project [5]. These included isolates of *P. rettgeri* and *P. alcalifaciens* as well as isolates of the species *Providencia stuartii* and *Providencia rustigianii* (Figure 6A)*.* These isolates will be referred to as the HMP isolates, to distinguish them from the Dmel isolates sequenced in our study. We used the 1651 orthologous genes found in all eight sequenced *Providencia* and *Proteus mirabilis* to determine the phylogenic relationship of these bacteria. We found that *P. stuartii* shared its most recent common ancestor with *P. burhodogranariea*, while *P. rustigianii* and *P. alcalifaciens* share their most recent common ancestor (Figure 6A). This phylogenic analysis also indicates that the genes found in the core genome of the two isolates of *P. alcalifaciens* are more similar to one another than the two *P. rettgeri* isolates (Figure 6A).

We hypothesized that the four HMP isolates might have specialized genes to facilitate living in the human gut while the Dmel isolates would have genes enabling infection of *D. melanogaster* and other insects. To test this hypothesis, we extracted all orthologs of the eight genomes, yielding 4926 total ortholog clusters. Only 177 orthologous clusters, 3.5% of the total, contained no genes from any HMP isolates and were therefore specific to the Dmel isolates. None of these contained genes found in all four Dmel isolates, meaning there are no genes that are exclusive to and universal in these isolates. Similarly, only 354, or 7.2% of the total ortholog clusters contain no genes from any Dmel isolate and therefore were exclusive to the HMP isolates. The majority of the HMP-specific ortholog clusters, 235 clusters, only contain two orthologous genes. Eleven of the HMP-specific ortholog clusters are found in all four of the isolates. Eight of these are assigned annotations that relate to phage activity and are physically co-localized in their respective genome. It is therefore unlikely that they are required for colonization of the human gut *per se*, but instead they probably reflect the shared phage pressure in the common environment. These isolates did not all originate from the same human, suggesting that the relevant phage may be pervasive in human guts. These phage genes were identified as belonging to Fels-2 in *P. alcalifaciens* HMP and the Myoviridae prophages PSP3 in *P. stuartii* HMP*, P. rettgeri* HMP*,* and *P. rustigianii* HMP*.* These two prophages are closely related to each other [58]. Of the 354 HMP isolate specific orthologs and paralogs, 69 of them are found only in *P. stuartii*. Most of these, 47, are annotated as hypothetical proteins. The total data indicate that there are no endogenous *Providencia* genes that are specific to and ubiquitous in either isolation environment we examined, but we do find evidence that the bacteria are exposed to different phages in the respective environments.

The core genome derived from all eight *Providencia* genome sequences contained 1925 genes, only very slightly fewer than the 1983 genes in the core genome of the Dmel *Providencia* isolates. This gives us added certainty that the core genome of *Providencia* as a whole is highly conserved. We estimate the asymptote of the core genome size for the genus *Providencia* as more genomes are sequenced will be around 1900 orthologs (Figure 6C).

The proportion of genes unique to any one of the HMP isolates is approximately the same as the unique gene complement in the Dmel strains. Unique genes represent 9% to 17% of the gene total for each of the isolates when all eight genomes are examined. Unsurprisingly, the HMP isolates of *P. alcalifaciens* and *P. rettgeri* share genes with only the Dmel isolates of these same species. Whereas 16% of the *P. alcalifaciens* Dmel genome consisted of unique genes when the Dmel isolates were considered alone, that value drops to 9% when the HMP isolates are also considered. The number of unique genes in *P*. *rettgeri* Dmel shows a similar decrease, from 19% of the total genes when Dmel isolates are considered in isolation to 10% after inclusion of the HMP isolates. However, these decreases in the count of unique genes are not only due to the addition of another isolate of the same species. Of the 800 genes initially considered unique to the Dmel isolate of *P. rettgeri,* 167 are found in the HMP isolates of *P. stuartii, P. alcalifaciens,* or *P. rustigianii.* Half of the decrease in apparently unique genes in *P. alcalifaciens* Dmel is due to orthologs in the HMP isolates of *P. stuartii, P. rettgeri* and *P. rustigianii.*

**Figure 9:**
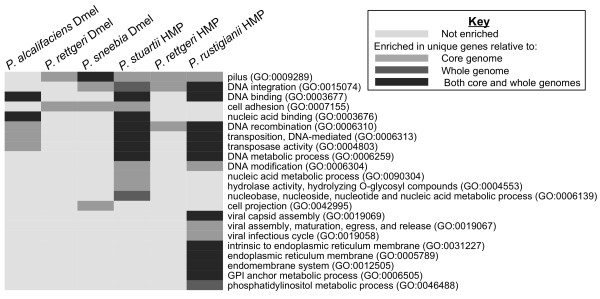
**GO terms enriched in the unique genes of each species when comparing the isolates collected from *D. melanogaster *(Dmel)to the isolates from the Human Microbiome Project(HMP).** GO terms were assigned and calculation of enrichment was done using a Fisher’s Exact Test using Blast2GO [24]. GO terms have been collapsed to only the most specific child term when multiple terms described the same group of genes. No GO terms were enriched in *P. burhodogranariea* Dmel or *P. alcalifaciens* HMP.

The unique, single copy genes for each of the eight genomes were assigned GO terms and then examined for GO term enrichment relative to either the whole genome or the core genome of all eight sequenced *Providencia* (Figure 9). Both the Dmel isolate of *P. burhodogranariea* and the HMP isolate of *P. alcalifaciens* had no GO terms enriched in their unique genes compared to either their respective whole genomes or the core genome. This suggests that the distinct genes acquired by these isolates have a wide variety of functions. There is also no shared enrichment for GO terms among all isolates originating from the human gut or among all those isolated from *D. melanogaster*, further emphasizing that there is no class of genes that tie the isolates together based on isolation environment. The unique genes *of P. rustigianii* HMP also are enriched in GO terms relating to the endoplasmic reticulum (ER) membrane compared to both the core and *P. rustigianii* HMP whole genome (Figure 9). These proteins each have a PGAP1-like domain, which is known to function in the ER, but since bacteria do not have an ER, they are unlikely to be functioning the same way in *P. rustigianii.* Although this domain is found in other bacteria, to our knowledge, no bacterial function has been determined for this domain.

GO categories related to transposons or transposition were enriched in several genomes, suggesting that the genomes have unique transposable elements (Figure 9). Additionally, *P. rustigianii* HMP unique genes have a number of GO terms related to viruses that are enriched over the core and whole genome (Figure 9). These genes are present in multiple locations throughout the genome and are either annotated as phage proteins or are surrounded by prophage genes. This, again, emphasizes the variety of prophages and other mobile elements inserted in these genomes.

As seen when considering the Dmel genomes alone, the HMP genomes are also enriched for unique genes with the GO term “pilus” relative to the core genome (Figure 9). The enrichment in the GO term “pilus” disappears from the Dmel isolates of *P. burhodogranariea* and *P. alcalifaciens* after addition of the HMP isolates because some of the genes with this GO term previously considered “unique” are additionally found in the HMP isolates. *P. sneebia* and *P. rettgeri* retain enrichment of the pilus GO term as well as other GO terms related to adhesion in their set of unique genes relative to the core genome, emphasizing the uniqueness of their fimbrial proteins compared to the other Dmel strains and the HMP strains.

### Species-specific genes

Two different isolates of both *P. rettgeri* and *P. alcalifaciens* have now been sequenced. One isolate of each species was sequenced from infected wild *D. melanogaster* (Dmel) while the other was isolated from human feces (HMP). There are 202 ortholog clusters that contain genes found only in the Dmel and HMP isolates of *P. rettgeri*, with six clusters containing more than two genes. The two isolates of *P. alcalifaciens* uniquely share 190 species-specific orthologous clusters. Unfortunately, most of these species-specific genes are annotated as hypothetical proteins so they do not lend any insight into biological distinction of these species from others in the *Providencia* genus. As described above for the individual isolates, genes annotated with the GO term “pilus” are enriched in genes specific to *P. alcalifaciens* relative to the *Providencia* core genome, and *P. rettgeri* is enriched for pilus genes and genes involved in rhamnose metabolic processes.

### Type 3 secretion systems

A type 3 secretion system (T3SS) is a needle-like apparatus used by Gram-negative bacteria for injecting effector proteins into host cells [59]. The genes encoding the proteins of the needle machinery are physically clustered and may be acquired via horizontal gene transfer as a single “pathogenicity island”. Transcription of the genes encoding the T3SS machinery and secretion of the effector proteins is triggered by external signals indicating that the bacteria is in the infection environment [60]. The functions of the translocated effector proteins vary greatly among bacteria, and include toxins that kill the host cells and proteins that manipulate host cell cytoskeletal activity or other cell biology to the advantage of the bacterium. Effector proteins are not necessarily encoded for within the same pathogenicity island as the genes encoding the T3SS needle apparatus, and may be acquired and evolve independently of the machinery [61].

**Figure 10:**
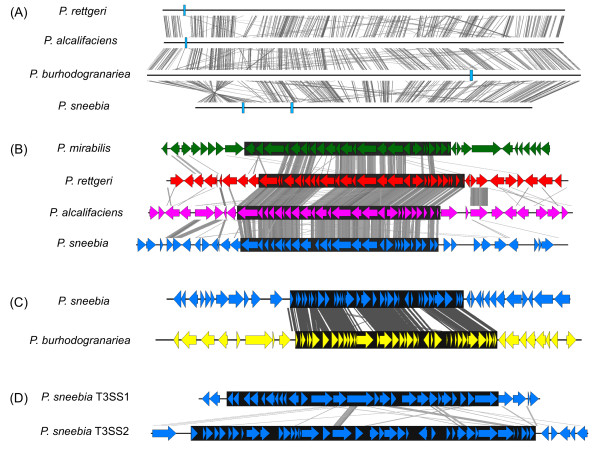
**Similarity of type 3 secretion systems(T3SS).** (**A**) Alignment of the whole genomes of *P. sneebia, P. rettgeri, P. alcalifaciens,* and *P. burhodogranariea* with the locations of each T3SS marked by blue boxes. (**B**) Alignment of T3SS-1 and some surrounding genes in *P. sneebia, P. rettgeri, P. alcalifaciens,* and *Proteus mirabilis*. Since this island falls within the inversion in *P. sneebia’s* genome*,* the region is displayed as the reverse-complement for ease of viewing. (**C**) Alignment of T3SS-2 and some surrounding genes in *P. sneebia* and *P. burhodogranariea*. (**D**) Alignment of T3SS-1 and T3SS-2 and some surrounding genes in *P. sneebia*. Figures were made using EasyFig [35]. Lines connecting the sequence schematics indicate regions of similarity, with darker grey indicating greater similarity. Colored arrows indicate individual genes and their direction. The black boxes behind the genes indicate the approximate boundaries of the T3SS island based on gene annotation.

All four *Providencia* sequenced in this study have at least one T3SS island (Figure 10A). *P. sneebia* Dmel*, P. alcalifaciens* Dmel*,* and *P. rettgeri* Dmel all have a similar T3SS island, which will be referred to as T3SS-1. These T3SS-1 islands are similar in sequence, gene content, gene orientation, and *ATPase* homology (Figure 10B). It is likely that this island was acquired prior to speciation of the sequenced *Providencia* as it is shared with *Proteus mirabilis* and the HMP *Providencia* isolates (Figure 10 A and B) and is found in the same syntenic region in all three Dmel genomes, although this position lies inside the *P. sneebia* inversion*.* The genomic region surrounding the T3SS-1 island is much less conserved than the genes of the island itself. Even though *P. sneebia, P. alcalifaciens,* and *P. rettgeri* all share this T3SS, these bacteria have been shown to vary in virulence towards *D. melanogaster*[18]*.* Some work has been done to characterize the T3SS-1 island of *Proteus mirabilis* during infection of the mouse ascending urinary tract, but disrupting the function of the secretion machinery had no effect on the bacteria’s ability to colonize the mouse [62].

There is a second T3SS island present in the *P. sneebia* genome that is also found in *P. burhodogranariea*. We refer to this as T3SS-2. The T3SS-2 island is not found in any of the *Providencia* HMP isolates. Although T3SS-2 is not syntenically conserved in its genomic location between *P. sneebia* and *P. burhodogranariea*, the sequences and gene contents of the islands from the two species are much more similar to each other than they are to T3SS-1 of *P. sneebia, P. alcalifaciens,* and *P. rettgeri*, or even than the respective T3SS-1 islands are to each other (Figure 10). It would be equally parsimonious to conclude that the *P. sneebia* and *P*. *burhodogranariea* genomes have separately acquired T3SS-2 islands or to infer a single acquisition by their common ancestor followed by a subsequent relocation of the island in one of the genomes.

The ATPases of T3SS-1 and T3SS-2 belong to different families. The T3SS-1 ATPase belongs to the Inv-Mxi-Spa ATPase family, which generally functions in cell invasion [61]. The ATPase of T3SS-2 belongs to the Ysc ATPase family, which is commonly found in extracellular pathogens [61]. Since *P. sneebia* carries both T3SS islands, it might be capable of functioning as both an extracellular pathogen and an intracellular one depending on infection context. However, the *ATPase* of T3SS-1 in our sequenced *P. sneebia* isolate contains a premature stop codon at codon 281 of the 420-codon gene, which likely abolishes the function of T3SS-1 in this isolate. We confirmed this stop codon by PCR and Sanger sequencing. We additionally sequenced the T3SS-1 *ATPase* from 8 additional isolates of *P. sneebia* originating from the hemolymph of wild caught *D. melanogaster* and found that they all have this stop codon (data not shown). While this does not mean all isolates of *P. sneebia* will have a stop codon in the *ATPase* of T3SS-1, it does suggest that this ATPase does not need to be functional for *P. sneebia* to cause an infection in *D. melanogaster.* None of the sequenced strains of *P. sneebia, P. rettgeri* and *P. alcalifaciens* showed evidence for the ability to intracellularly replicate in *D. melanogaster* cells *in vitro*, although *P. alcalifaciens* showed some evidence for being invasive and *P. rettgeri* and *P. sneebia* had evidence for resisting phagocytosis [18]. While a T3SS may be involved in these phenotypes for each species, it remains possible that these bacteria may have different intracellular replication or invasive phenotypes in *D. melanogaster in vivo* or in other hosts. Indeed, different strains of *P. alcalifaciens* have previously been shown to exhibit invasion of vertebrate cells [63,64].

## Conclusions

We sequenced and compared the draft genomes of four species of *Providencia* that were all isolated from the hemolymph of wild caught *D. melanogaster*[17,18]*.* We found the core genome of these isolates to be about 60% of the total coding content for each genome, even after inclusion of four isolates of *Providencia* originating from human feces. We found no genes that were specific to and universal in bacteria isolated either from *D*. *melanogaster* or from the human gut. Approximately 15% of each genome sequence consisted of genes unique to that isolate. These genes should explain variable phenotypes among the isolates, including metabolic differences [17] and variation in virulence towards *D. melanogaster*[18]*.* We found that each of these isolates has at least one type 3 secretion system. The unique genes of each genome are enriched for genes with the “pilus” GO term, suggesting variation in substrates that the bacteria adhere to. The T3SSs and the variety of adhesion molecules suggest that host cell contact is an important part of the virulence mechanisms for these *Providencia*.

## Abbreviations

ORF: Open reading frame; GO: Gene ontology; FDR: False discovery rate; Dmel: Bacteria isolated from hemolymph of *D. melanogaster*; HMP: Bacteria sequenced by Human Microbiome Project; T3SS: Type 3 secretion system.

## Competing interests

The authors declare that they have no competing interests.

## Authors’ contributions

MRG and BPL conceived of the study, participated in its design, and drafted the manuscript. MRG preformed all computational work and PCRs. All authors read and approved the final manuscript.

## Supplementary Material

Additional file 1 **Figure S1. **Flow chart illustrating steps taken in the assembly of the *P. sneebia* and *P. rettgeri* genomes. Click here for file
