# Supplementary figures and images for: Comparative genomics of bacteria in the genus Providencia isolated from wild Drosophila melanogaster
Source: BMC Genomics. 2012 Nov 13;13:612. doi: 10.1186/1471-2164-13-612 (PMC3542290; doi:10.1186/1471-2164-13-612)

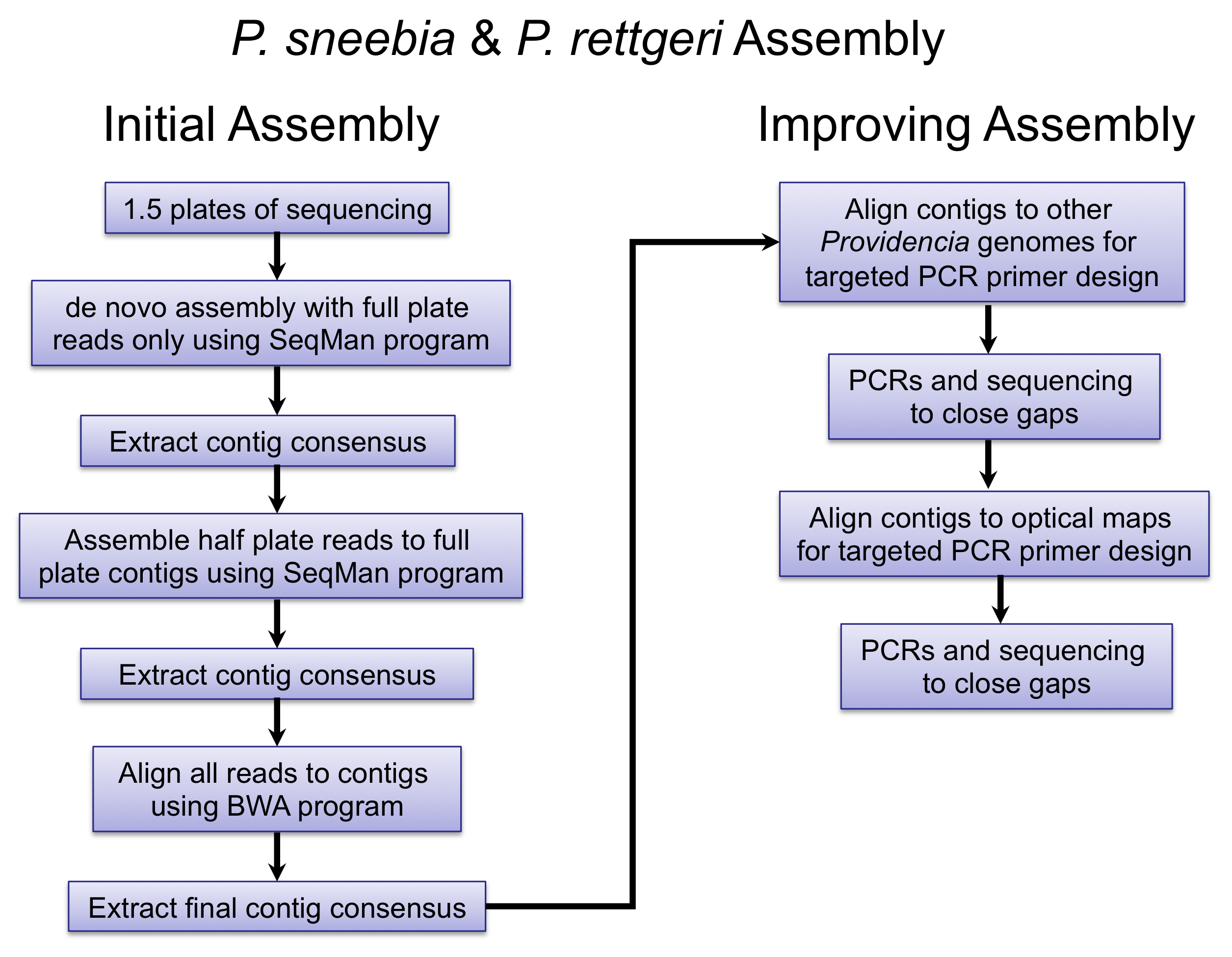

Supplement: Additional file 1 — Figure S1. Flow chart illustrating steps taken in the assembly of the P. sneebia and P. rettgeri genomes. [file 1471-2164-13-612-S1.tiff]
